# Development of a Real-Time Thermoplastic Mask Compression Force Monitoring System Using Capacitive Force Sensor

**DOI:** 10.3389/frobt.2022.778594

**Published:** 2022-07-06

**Authors:** Tae-Ho Kim, Min-Seok Cho, Dong-Seok Shin, Dong Ho Shin, Siyong Kim

**Affiliations:** ^1^ Proton Therapy Center, National Cancer Center, Goyang, South Korea; ^2^ Department of Radiation Oncology, Yongin Severance Hospital, Yongin, South Korea; ^3^ Department of Radiation Oncology, Virginia Commonwealth University, Virginia, VA, United States

**Keywords:** thermoplastic mask head fixation, real-time motion control, head and neck cancer, motion prediction, capacitive force sensor

## Abstract

**Purpose:** Thermoplastic masks keep patients in an appropriate position to ensure accurate radiation delivery. For a thermoplastic mask to maintain clinical efficacy, the mask should wrap the patient's surface properly and provide uniform pressure to all areas. However, to our best knowledge, no explicit method for achieving such a goal currently exists. Therefore, in this study, we intended to develop a real-time thermoplastic mask compression force (TMCF) monitoring system to measure compression force quantitatively. A prototype system was fabricated, and the feasibility of the proposed method was evaluated.

**Methods:** The real-time TMCF monitoring system basically consists of four force sensor units, a microcontroller board (Arduino Bluno Mega 2560), a control PC, and an in-house software program. To evaluate the reproducibility of the TMCF monitoring system, both a reproducibility test using a micrometer and a setup reproducibility test using a head phantom were performed. Additionally, the reproducibility tests of mask setup and motion detection tests were carried out with a cohort of six volunteers.

**Results:** The system provided stable pressure readings in all 10 trials during the sensor unit reproducibility test. The largest standard deviation (SD) among trials was about 36 gf/cm^2^ (∼2.4% of the full-scale range). For five repeated mask setups on the phantom, the compression force variation of the mask was less than 39 gf/cm^2^ (2.6% of the full-scale range). We were successful in making masks together with the monitoring system connected and demonstrated feasible utilization of the system. Compression force variations were observed among the volunteers and according to the location of the sensor (among forehead, both cheekbones, and chin). The TMCF monitoring system provided the information in real time on whether the mask was properly pressing the human subject as an immobilization tool.

**Conclusion:** With the developed system, it is possible to monitor the effectiveness of the mask in real time by continuously measuring the compression force between the mask and patient during the treatment. The graphical user interface (GUI) of the monitoring system developed provides a warning signal when the compression force of the mask is insufficient. Although the number of volunteers participated in the study was small, the obtained preliminary results suggest that the system could ostensibly improve the setup accuracy of a thermoplastic mask.

## 1 Introduction

For accurate delivery of radiation therapy, daily setup accuracy and reproducibility are critical, especially for high-precision techniques like intensity-modulated radiotherapy (Purdy, 2000; Karger et al., 2001). In head and neck radiation therapy, thermoplastic mask systems have been widely used, and there are ample studies on their effectiveness and efficiency in reducing patient setup errors (Willner et al., 1997; Sweeney et al., 1998; Tsai et al., 1999; Gilbeau et al., 2001; Fuss et al., 2004).

A thermoplastic mask is made of a plastic material and is mesh-like. The mask holds the head and neck still and in the exact position needed for radiation treatment. It makes the patient in a position to ensure accurate delivery of the radiation beam and helps ensure that the setup position is as accurate and as effective as possible. A typical procedure for using the thermoplastic mask is as follows:

1) Place an unformed thermoplastic mask in a heater (for example, a wet or dry bath) with the set temperature and time to make the mask flexible.
2) Take the mask out of the bath. In case of using a water bath quickly remove excess water for patient comfort.
3) Check if the temperature of the mask is not too hot so it can be safely placed on the patient skin.
4) Position the mask on the patient face. Align the nose hole of the mask with the patient nose.
5) Mold the mask precisely around the nose bridge, chin, and eye sockets of the patient.
6) When the facial features have been precisely molded into the mask, start cooling down the mask.
7) When completely cooled, remove the mask.

Although these systems provide effective immobilization capabilities to a certain extent (Boda-Heggemann et al., 2006; Tryggestad et al., 2011), it is not unusual for them to suffer from noticeable daily variations in target alignment, especially when there are patient contour changes. Many studies, for this reason, have pointed out that it is not easy to perform accurate patient setups with a thermoplastic mask only. Therefore, it is recommended to use additional monitoring mechanisms such as x-ray-based guidance systems (for example, electronic portal imaging device, kV planar imaging, and/or cone-beam computed tomography) to reduce setup errors (Karger et al., 2001; Hong et al., 2005; Tae-Ho et al., 2012).

However, x-ray-based monitoring has a potential risk from excessive radiation exposure and no feasibility of monitoring a patient during the treatment (Li et al., 2006). In order to avoid excessive radiation exposure, optical camera-based monitoring systems have been developed (Buatti et al., 1998; Wagner et al., 2007), but such systems actually monitor the thermoplastic mask––not the patient skin directly (Kim et al., 2004; Linthout et al., 2006).

For thermoplastic masks to maintain clinical efficacy, they should wrap the patient surface well and apply appropriate pressure to all areas (Orfit Industries, 2022, Instructions for Use). Also, if possible, the compression force of the mask is desired not to change through the whole treatment period. However, it may become inconsistent due to various causes such as deformation of the mask itself, patient contour change, and motion. These changes can reduce the ability of the mask as an immobilization tool and increase the magnitude of patient positioning error. Therefore, it is desirable to quantify the compression force of a mask and maintain proper compression force throughout the entire procedure to our best knowledge; however, no explicit and widely accepted method for achieving such a goal currently exists.

In a previous study, our research group performed basic tests on whether real-time compression force monitoring could be useful for quality assurance (QA) of immobilization tools used in radiation therapy. Through the study, a new monitoring method was proposed (a method different from the existing x-ray- or camera-based monitoring), and the possibility of improving patient setup accuracy was presented (Cho et al., 2016). However, force sensors made of polymer materials that had been used in the previous study turned out being suffered from the drift that could last several minutes (increasing/decreasing trends of sensor output in response to constant stimulus).

In this study, therefore, we intended to develop a real-time thermoplastic mask compression force (TMCF) monitoring system using a capacitive force sensor that showed a small drift and was suitable for force monitoring in the human body (Tang et al., 2020). A prototype system was fabricated, and the feasibility of the proposed method was evaluated.

## 2 Materials and Methods

### 2.1 Real-Time TMCF Monitoring System

**FIGURE 1:**
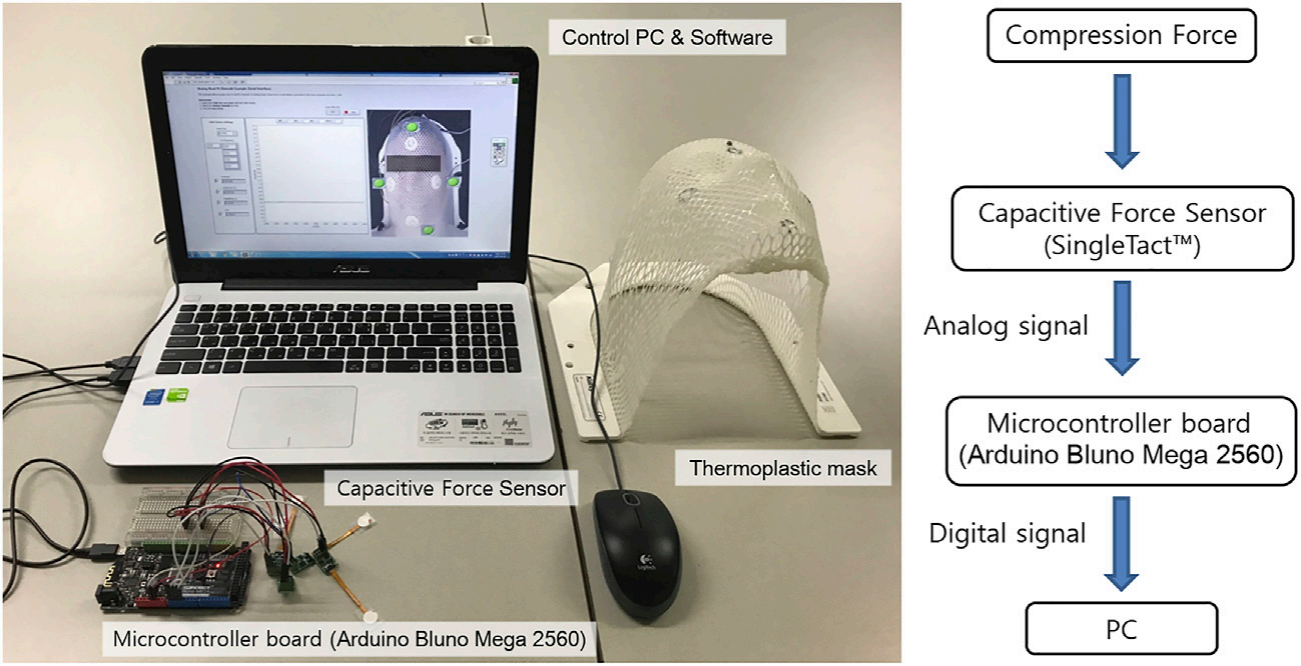
Real-time TMCF monitoring system includes a force sensor, a microcontroller board, a control system PC, and data acquisition software.

**FIGURE 2:**
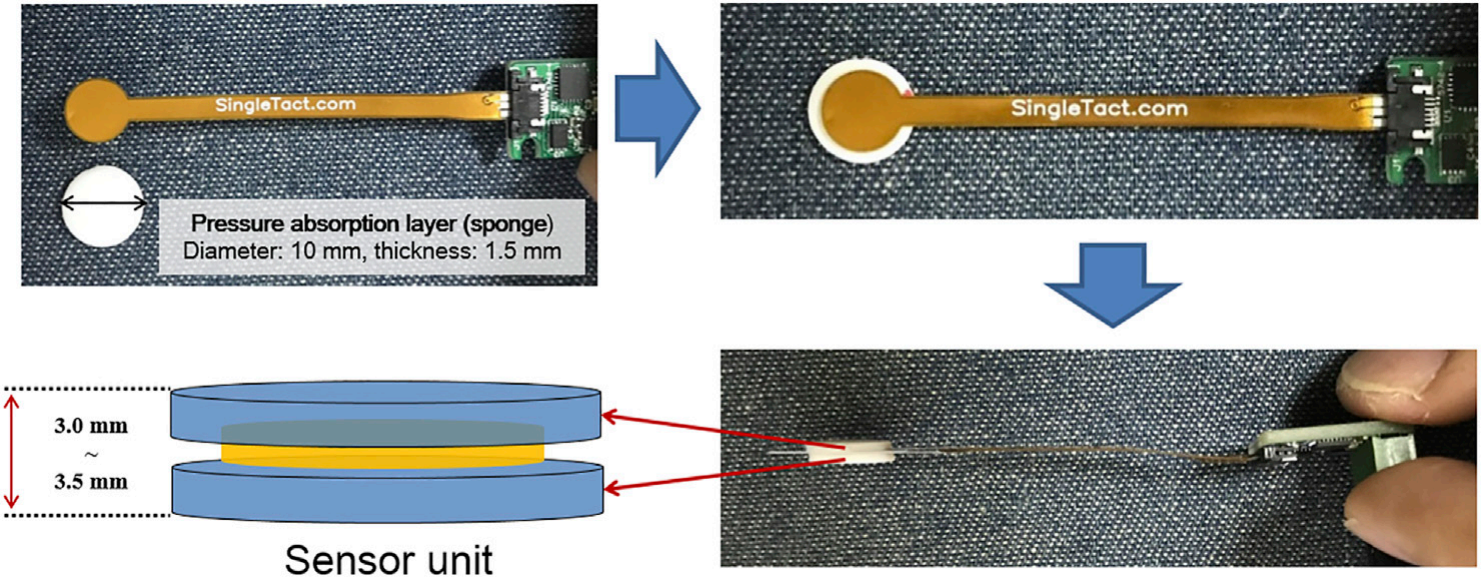
Sensor unit consists of a force sensor and pressure absorption layer (PAL). The total thickness of the sensor unit in this study is 3.0–3.5 mm.

The real-time TMCF monitoring system basically consists of four force sensor units, a microcontroller board (Arduino Bluno Mega 2560), a control PC, and an in-house software program (Figure 1). In this study, a SingleTact™ capacitive force sensor manufactured by Pressure Profile System, Inc. (Glasgow, UK) was used. SingleTact™ is a capacitive force sensor with a parallel capacitive configuration, and it is considered to be suitable for this application due to its high accuracy and repeatability (Tang et al., 2020). SingleTact™ consists of two thin round polyimide electrode plates separated by a dielectric. The working principle of the sensor is based on flexible interlayer compression as a result of the force that arises from distance change between two electrodes causing capacitance variation (Graudone et al., 2020). SingleTact™ includes electronics providing an interface to the main controller. The supply voltage needs to be between 3.7 V and 12 V with an input current of 2.7 mA. The range of the analog output voltage is between 0.5 V and 1.5 V. In terms of data transfer, SingleTact™ can accommodate more than 100 Hz (Warsito et al., 2020). For real-time TMCF monitoring, a calibrated sensor CS8-10N with a diameter of 8 mm was used. Calibrated sensors offer improved accuracy and linearity over standard sensors and come as a matched sensor plus electronics interface board providing a pre-configured system in terms of the electronic interface (Figure 2.) and with calibration carried out for linear output. Pre-calibrated sensors can measure pressure up to over 1500 gf/cm^2^ with a resolution of about 0.2% of the full scale. The response time of the system is 1 ms (Pressure Prole Syst., Inc, 2022a, User Manual).

While the thickness of the sensor itself is 0.35 mm, two sponge pads of about 3 mm (that is, 1.5 mm each), called pressure absorption layer (PAL), are added, one above and the other below, to improve contact sensitivity between the mask and patient skin as shown in Figure 2. A thermoplastic mask, in an ideal situation, should be formed tightly to the patient skin without gaps. In reality, however, there are often small gaps (about 1 mm). Therefore, an appropriate thickness of PAL can help improve the contact sensitivity between the mask and the patient skin. The PAL thickness needs to be adjusted depending on the position to be monitored and the degree of contact between the mask and the face. It is also instrumental to select an appropriate material for PAL.

Initially, we tried to use silicon material (Dragon Skin TM 30) with stiffness similar to that of skin (30 Shore A). However, its stiffness turned out too strong to be used for mask force monitoring (the sensor signal got saturated easily). Therefore, the PAL material was changed to a soft sponge (about 60 Shore OO, stiffness similar to that of sliced bread). In order to find an appropriate PAL thickness that can reliably measure the mask force within the active range of the sensor, the thickness of the sponge was increased by a 1-mm interval.

When the PAL is too thin (1 mm), as compared to the optimal value (3 mm), movements inside the mask would not be monitored effectively. When the PAL was 1 mm, TMCF baseline values were observed between 80 and 120 gf/cm^2^. As can be seen in Figure 5, this value is the force when the soft sponge (PAL) is compressed by about 0.1 mm. Considering the stiffness of the soft sponge, it can be seen that the value is an insufficient force to keep the TMCF stable.

Actually, in real-time monitoring conducted on six volunteers, it was confirmed that the lower the baseline of TMCF, the more difficult it is to maintain the compression force stable. In addition, it has been confirmed that if the TMCF falls below 100 gf/cm^2^ (that is, when TMCF is too low), the compression force can no longer be maintained and often drops to zero. In other words, 100 gf/cm^2^ seemed a threshold value when the mask was about to lose meaningful contact with the surface of the volunteer. Therefore, the value of 100 gf/cm^2^ was applied as the first tolerance. The current version of GUI displays a yellow light warning to alert the user when the real-time TMCF falls below 100 gf/cm^2^ (first tolerance). Also, if TMCF drops to ‘0' (2nd tolerance), it is designed to give a red-light warning.

In this study, every mask was made by a single experienced radiation therapy technologist to keep consistency and about 3 mm of PAL was found appropriate in effectively monitoring mask force. When the PAL is too thick (over 5 mm PAL), as compared to the optimal value (3 mm), the sensor would encounter frequent signal saturation. Thus, the total thickness of the force sensor unit (sensor and the optimal value of PAL) is about 3.35 mm (0.35 + 3 mm). Note, in actual practice, the optimal PAL thickness may vary depending on what type of mask is used and how it is formed.

The sensor units were placed between the thermoplastic mask and the patient face at four sensing points (forehead, both cheeks, and chin). It is recommended to avoid bending a sensor if possible because when bent, one electrode would be in compression while the other in tension, which can cause shearing to occur between the two electrodes. When bending is unavoidable, it is recommended to keep the circular portion (the active sensing region) as flat as possible while applying the bend along the tail end of the sensor. It is also necessary to keep a bend radius greater than 3 mm (Pressure Prole Syst., Inc, 2022b, SingleTact Mounting Methods). Therefore, caution is needed when placing sensors, especially on the curved parts of the patient face.

The analog signal produced by the capacitive sensor is digitized with an Arduino Bluno Mega 2560 and connected to a PC for data processing and analysis (Figure 1). The in-house developed software program, written in LabView, continuously monitors compression forces at four points and performs data processing to indicate whether the pressure at each point is within tolerance or not.

### 2.2 Reproducibility Test of the Sensor Unit

**FIGURE 3:**
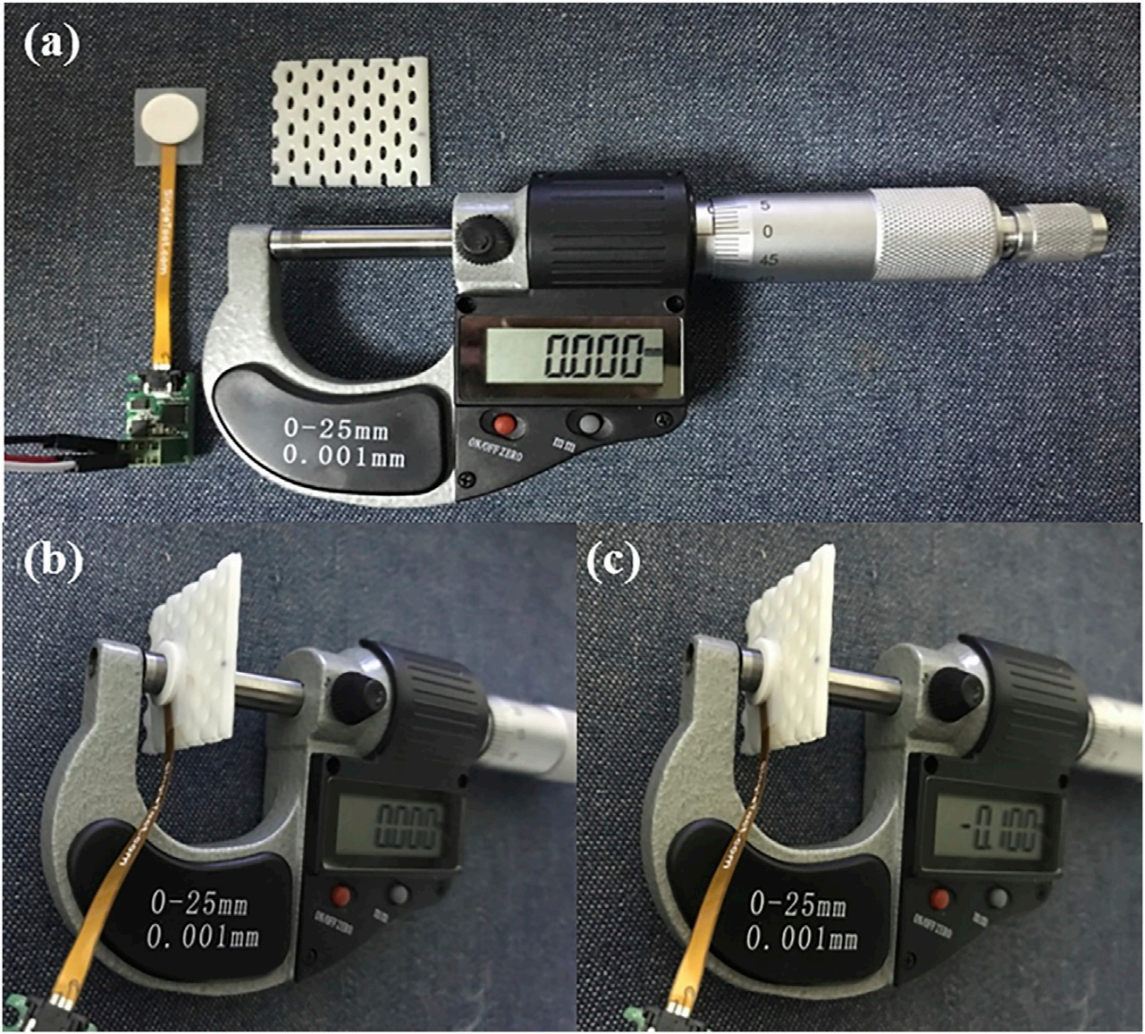
Experimental setting for the reproducibility test of the sensor unit. **(A)** Prepare materials for the test, the sensor unit, a piece of thermoplastic mask, and a micrometer; **(B)** place the sensor unit and the piece of mask between the anvil and spindle of the micrometer, and then set the scale to zero; **(C)** obtain sensor output values according to different compressing distances with the interval of 0.1 mm.

In this test, we tried to evaluate whether the same pressure values were obtained repeatedly when the sensor unit was pressed with known forces. A micrometer with a resolution of 0.001 mm was used to compress the PAL consistently. Figure 3 shows the experimental setting for the sensor unit reproducibility test. The procedure is as follows:

1) Place a piece of mask and the sensor unit between the anvil and spindle of the micrometer (Figures 3A,B).
2) Adjust the distance between the anvil and spindle of the micrometer to the extent that the sensor signal is not measured.
3) Set the micrometer scale to zero (Figure 3B).
4) Obtain data (sensor output values) according to different compressing distances with the interval of 0.1 mm (Figure 3C).

Sensor output was recorded every 100 milliseconds for 1 min in each trial, and a total of 10 trials were made for all four sensor units.

### 2.3 Effect of Time on the Measurement Accuracy Test and Reproducibility Test of the Real Time TMCF Monitoring System With a Head Phantom

Constant compression was given for 20 min (a time interval comparable to a typical radiation therapy session), and pressure change with time was measured.

**FIGURE 4:**
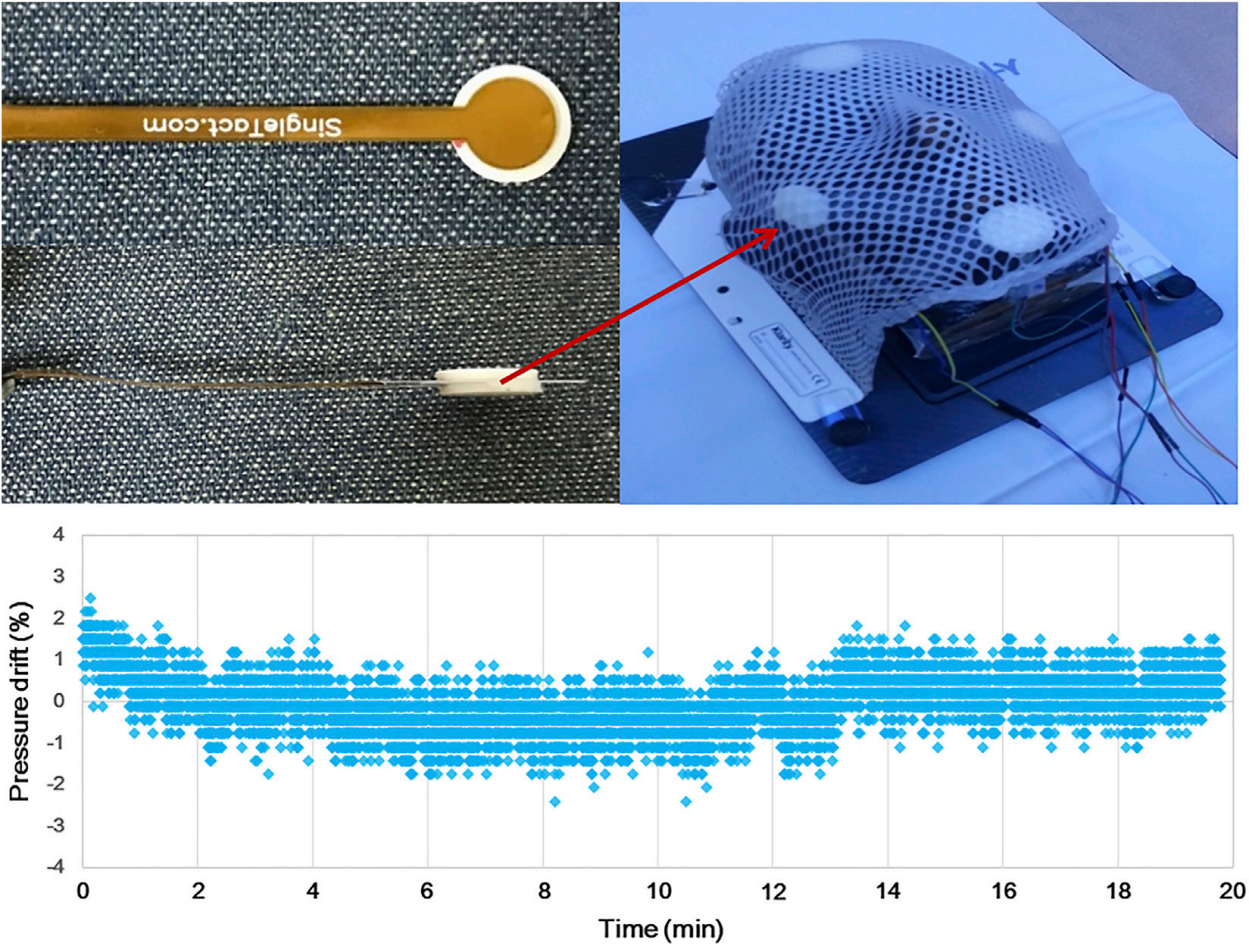
Experimental setting for both the time effect and reproducibility test of the real-time TMCF monitoring system. A total of four sensors were attached to the face of a head phantom (forehead, both cheeks, and chin), and then, the phantom was immobilized by a thermoplastic mask.

In addition, a reproducibility test was performed to evaluate the reliability of the developed monitoring system prior to a volunteer test. Under an ideal condition, compression force inside the mask is expected not to vary in multiple uses. To closely simulate an ideal situation, a stability test was performed with a rigid head phantom. Figure 4 shows the experimental setting for the system reproducibility test. Through the test, it was tried to confirm whether the same compression force was obtained repeatedly when the rigid head phantom was immobilized with the system in multiple trials. In each trial, the data were acquired every 100 milliseconds for 3 min (that is, 1800 samples for each trial), and then the mean value was recorded. This process was repeated for a total of five times, and the mean SD value was obtained.

### 2.4 Thermoplastic Mask Setup Reproducibility Study Using the Developed Real-Time TMCF Monitoring System With Volunteers

A total of six healthy volunteers (five males and one female) were enrolled in this simulation test. The volunteer group included two medical physicists, three graduate students, and one from the general public. The age ranged from 26 to 41 yrs.

For each volunteer, a thermoplastic mask was formed, and four force sensor units were attached to the protrusions of the face (that is, forehead, both cheekbones, and chin) using a transparent adhesive film (Tegaderm Film, 3M). After completing the mask setup, compression force values were monitored for 3 min in real time, which was considered one session. Each volunteer took four sessions, and, in between, the mask was off and an interval of approximately 10 min was given.

### 2.5 Compression Force Variation due to Motion Inside the Thermoplastic Mask (Simulation Study: Intended Motion Test)

To investigate the effectiveness of the system for detecting abrupt force/motion, volunteers were instructed to try to move intentionally to simulate situations where a relatively large force of motion and/or variation occurred within the mask. Attempts of intentional movement (for example, nod motion with short bursts of force) were made at 3 specific times (100, 300, and 500 msec after monitoring started). Compression force values were observed for 1 min and compared with the results of the previous test (that is, Section 2.4).

## 3 Results

### 3.1 Reproducibility Test of the Sensor Unit

Table 1 shows pressure values according to the compressed distance of the force sensor unit. In this test, we evaluated how consistent the monitored pressure values were among 10 trials. While absolute SD values tended to increase as the units were compressed more, relative ones decreased down to about 2.4% (that is, about 36 gf/cm^2^) of 1500 gf/cm^2^ (the max of full scale). Overall, less than about 5% of SD was observed with pressure values larger than approximately 300 gf/cm^2^. It was also found that pressure reading got almost saturated when the units were compressed to 1.1 mm or more.

**TABLE 1:** Summary of pressure values according to the compressed distance of force sensor units.

Compressed distance [mm]	Mean ± SD [gf/cm^2^]			
	Sensor # 1	Sensor # 2	Sensor # 3	Sensor # 4
0	0	0	0	0
0.1	87 ± 8	87 ± 9	85 ± 8	99 ± 7
0.2	152 ± 8	160 ± 10	162 ± 15	160 ± 9
0.3	250 ± 10	264 ± 11	259 ± 13	257 ± 15
0.4	378 ± 9	395 ± 16	377 ± 11	385 ± 14
0.5	493 ± 16	529 ± 15	515 ± 17	503 ± 20
0.6	650 ± 24	703 ± 19	694 ± 27	673 ± 21
0.7	849 ± 30	899 ± 21	874 ± 26	889 ± 27
0.8	1045 ± 35	1089 ± 28	1055 ± 30	1098 ± 33
0.9	1253 ± 32	1292 ± 30	1270 ± 29	1324 ± 31
1.0	1441 ± 36	1514 ± 27	1456 ± 33	1513 ± 36
> 1.1	1534.2	1586	1552	1579

**FIGURE 5:**
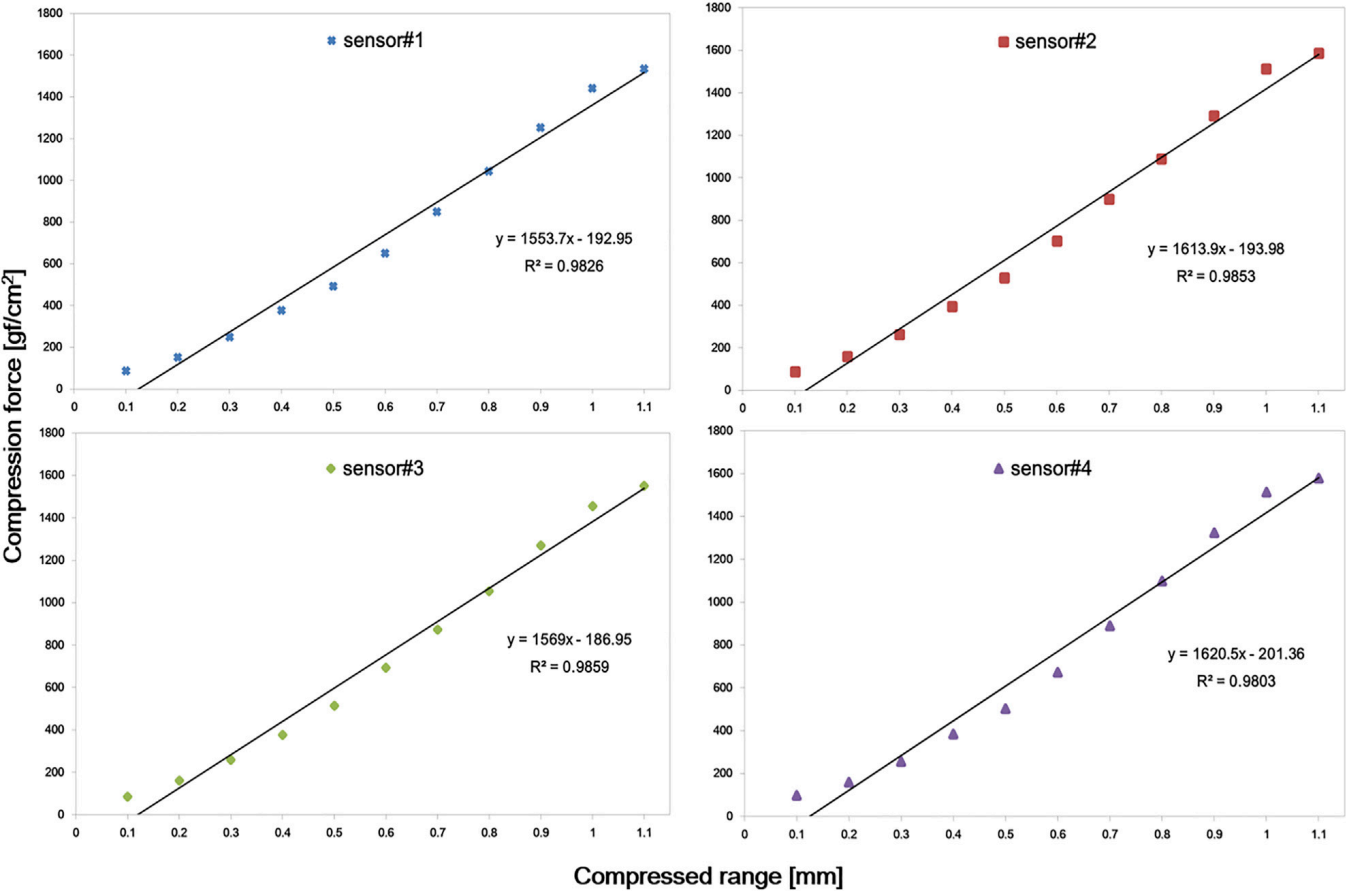
Plot of pressure values according to compression distances. A linear trend was observed in all four sensors within 1 mm compression.

Mean values in Table 1 are plotted according to compression distances in Figure 5. As it is shown in the figure, it could be observed that the differences between the measured forces for corresponding compressions increase with the compression since the stiffness of the PAL sponge increases as it is compressed (even if the PAL was compressed evenly to 1 mm, the pressure applied to the sensor gradually increased). However, as illustrated, a clear linearity between the obtained pressure and applied compression was obtained (R^2^ value over 0.98 obtained for all sensors).

### 3.2 Effect of Time on the Measurement Accuracy Test and Reproducibility Test of the Real-Time TMCF Monitoring System With a Head Phantom

The pressure drift of the SingleTact sensor fluctuates between the zero points, thus giving positive and negative results intermittently. No obvious trend of the pressure drift can be observed (Figure 4).

**FIGURE 6:**
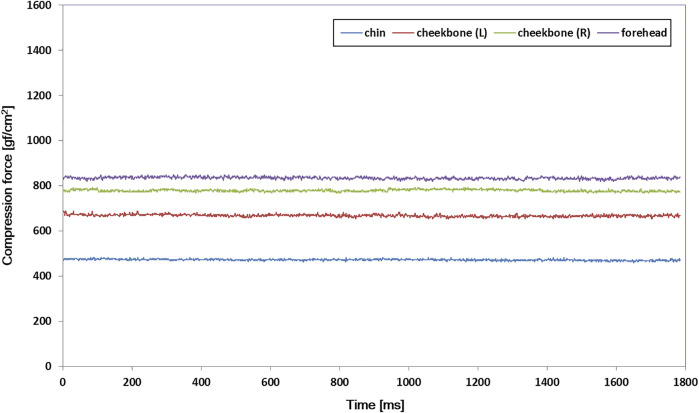
Compression force variations at four measuring spots inside the thermoplastic mask with a rigid head phantom for a 3-min trial. The output signal was maintained stable in all of the sensors.

In the time effect test with the head phantom, since there was neither contour change nor motion, constant compression forces of the mask were expected, and the system showed stable readings as shown in Figure 6.

Table 2 shows the results of the mask setup reproducibility test in which a total of five 3-min trials were repeated using the rigid head phantom. Because setups were attempted with the rigid object, if a precise mask setup was performed, compression force variation should be minimal. As summarized in Table 2, the standard deviation of each measuring spot did not exceed 39 gf/cm^2^ (2.6% of 1500 gf/cm^2^).

**TABLE 2:** Result summary of the thermoplastic mask setup reproducibility test with a rigid head phantom. In each trial, the data were acquired at every 100 ms for 3 min, and the data represent the mean values of 1,800 samples for each fraction.

Fraction	Pressure value [gf/cm^2^]			
	Forehead	Left cheekbone	Right cheekbone	Chin
1	876	715	814	494
2	893	698	731	454
3	834	641	792	417
4	818	731	756	506
5	802	655	824	503
Mean ± SD	844.6 ± 38	688 ± 39	783.4 ± 39	474.8 ± 38

### 3.3 Thermoplastic Mask Setup Reproducibility Study Using the Developed Real-Time TMCF Monitoring System With Volunteers

**FIGURE 7:**
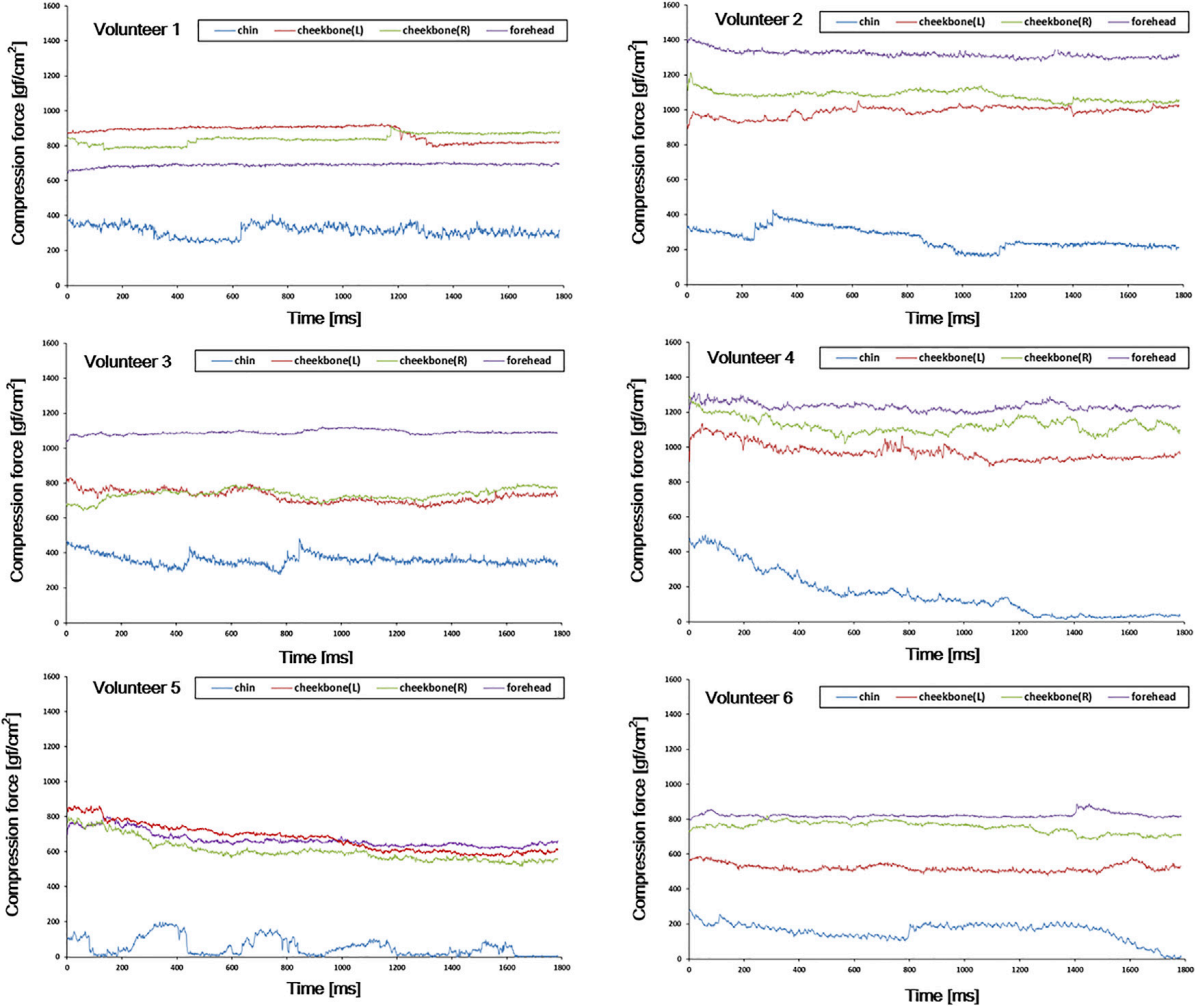
Compression force variation of each volunteer in the first 3-min session. Various compression force variations were observed depending on the location of the sensor (forehead, both cheekbones, and chin) and volunteers.

Figure 7 shows the compression force variation of each volunteer (result of the first session). Volunteers were pressed using a customized mask, and then the compression force variation was monitored for 3 min per session for four sessions. As can be seen in the figure, it was observed that the compression force varied in a noticeable amount with time in general unlike the cases of the previous test with the head phantom (Figure 6). The compression force of the forehead was generally higher and more stable than that of the other parts in most volunteers. Compression force values for both cheekbones were also generally stable in all volunteers. However, in the case of the chin, the overall compression force was lower than that in other regions. In addition, its variation was more dramatic and sometimes reached down to zero (0).

**FIGURE 8:**
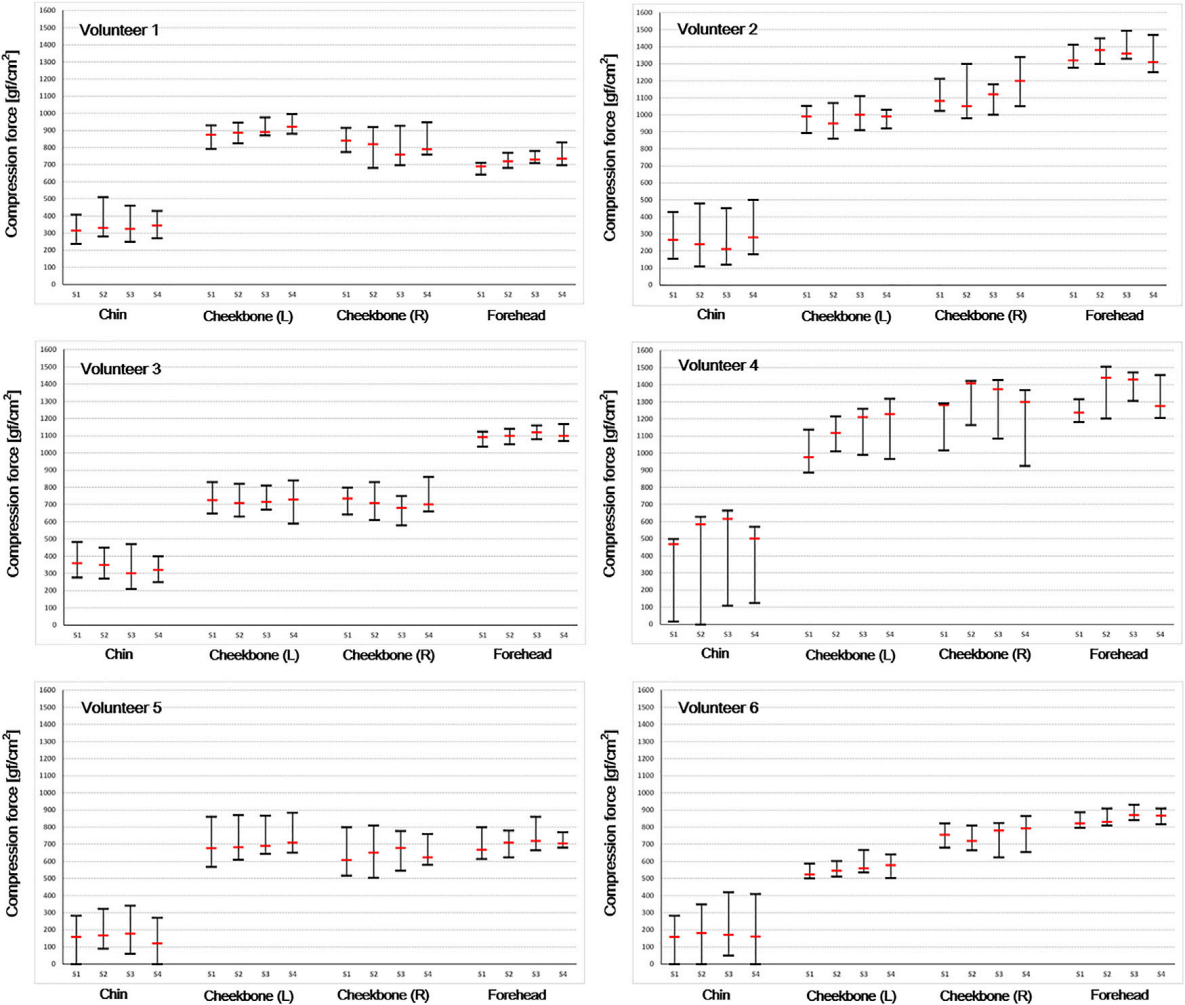
Compression force variation range and mean value were acquired to evaluate thermoplastic mask setup reproducibility. Each volunteer took four sessions (S1–S4 means each session number).

Figure 8 shows the variation range and mean value of compression force for each session. Volunteers 1, 2, 4, and 6 maintained high compression forces (>800 gf/cm^2^) at all sessions, and the compression forces of volunteers 3 and 5 also did not drop below 600 gf/cm^2^. In the case of both cheekbones, the variation ranges were slightly larger than those of the forehead case, but all volunteers maintained high compression forces in general (over 500 gf/cm^2^) in all sessions. In the chin case, the mean values were lower than those of other sites, and the ranges were noticeably larger in general. However, variations among sessions were not significant.

### 3.4 Compression Force Variation due to Motion Inside the Thermoplastic Mask (Simulation Study: Intended Motion Test)

**FIGURE 9:**
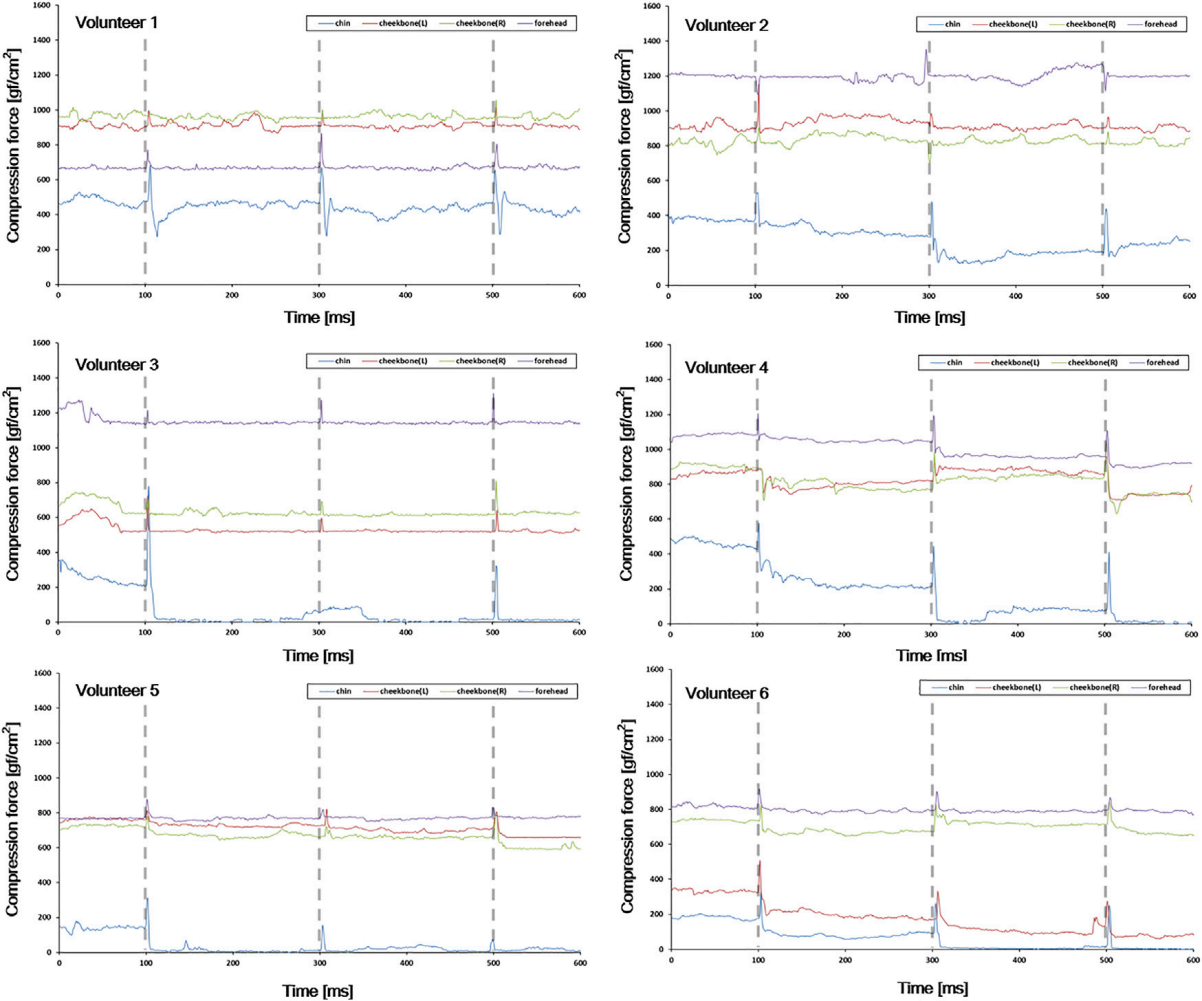
Compression force variation due to intended motion inside the thermoplastic mask. The gray vertical dotted line indicates the time at which the intended motion was instructed (10, 30, and 50 s after monitoring starts).

Figure 9 shows compression force variation due to intended motion inside the thermoplastic mask. As shown, noticeable changes in the compression force were observed at times when movement was instructed (that is, at 100, 300, and 500 ms), indicating the capability of the system to monitor possible patient motion and/or displacement inside the mask.

As mentioned previously, the GUI of the monitoring system is designed to give a yellow light warning signal when the real-time compression force value falls below 100 gf/cm^2^. Also, if the compression force value drops to “0”, it is designed to give a red-light warning signal. Therefore, if the yellow light is on, the patient should be observed more carefully, and if the red light is on, a readjustment is recommended because it can be assumed that the contact between the mask and face is not appropriate.

## 4 Discussion

The thermoplastic mask is considered an effective immobilization tool that can prevent patients from movement (Willner et al., 1997; Sweeney et al., 1998; Tsai et al., 1999; Gilbeau et al., 2001; Fuss et al., 2004). This clinical utility assumes that the compression force inside the mask is maintained without significant change during the treatment period. On the other hand, it is commonly agreed that the compression force inside the mask may vary during the treatment period due to various causes such as patient contour change, patient motion, and deformation of the mask itself. Nevertheless, such assumption that consistent compression force is maintained inside the mask has never been actively confirmed on a real-time basis. We believe this is mainly because there has been no system available that can provide relevant information (for example, compression force) in real time. We took a challenge to develop such a system and performed a feasibility study of the system with a cohort of volunteers in this study.

In this study, PAL was added above and below the sensor itself to suit better the compression force monitoring inside the thermoplastic mask. As PAL was added, it was confirmed that the linearity of the sensor was slightly lower than the inherent performance of the sensor. Although there was concern that PAL would adversely affect the inherent performance of the sensor, as shown in Figure 5, it seems that there is no problem in monitoring the compression force of the mask (R^2^ value over 0.98 was obtained for all sensors within 1 mm compression).

However, since the thermoplastic mask is manufactured differently for each patient, in order to further improve the accuracy and usability of the system, further study about the optimization of PAL material, shape, and thickness may be necessary. For example, using a dome-shaped mechanical coupler attached to the sensor improved the transfer of forces from multiple directions to the active area of the sensor (Andreozzi et al., 2020; Andreozzi et al., 2021a; Andreozzi et al., 2021b). A design (for example, truncated cone shape) that can effectively place compression force to the center of the sensor area may also be a good solution.

Through the thermoplastic mask setup reproducibility test, we confirmed the reliability of the developed monitoring system (Table 2) prior to a volunteer test. We also confirmed that the developed monitoring system could keep the output signal stable if there was no change inside the mask (Figure 6).

As shown in Figure 7, various compression force changes were observed depending on the location of the sensor (that is, forehead, both cheekbones, and chin) and volunteer. In case of forehead and both cheekbones, the compression force was generally stable in all volunteers. This means that the mask limits the movement of the forehead and both cheekbones by relatively constant and large forces (forehead ≥600 gf/cm2, chin ≥500 gf/cm2). Such variation trend was observed in all four sessions (Figure 8). Also, in the simulation test performed assuming significant moving forces inside the mask, the baseline of the compression force did not significantly change or drop to zero before and after the peak value. In the case of the chin, the baseline of the compression force was lower than that of the other sites, and especially with volunteers 4, 5, and 6, the compression force often dropped close to zero (Figure 7, chin case). A “zero” compression force means that there is a gap between the mask and the patient surface, and the mask could no longer play a proper role as an immobilization tool. Such behaviors were consistent through multiple sessions as shown in Figure 8. Compression force dropping to “0” was more prominent in the intended motion test (Figure 9), implying that either the chin area is a weak point in mask fabrication or more moving force is given to the chin area by patients or both. Therefore, using more caution on the chin area may be necessary, and close monitoring would be highly beneficial.

The aforementioned TMCF variation trend was limited to the volunteers who participated in this study. It was difficult to generalize these results since the number of volunteers participating in the study was not large enough. The TMCF baseline and variation trend can be affected by various factors such as the skill of the radiologist making the mask, the characteristics of the patient surface, whether the patient can maintain the setup without moving, and the location of the sensor. In addition, since the patient is not a rigid object, it is natural for TMCF to change in real time.

TMCF variation does not necessarily mean that the patient setup position has changed. If the TMCF baseline is maintained above a certain value (100 gf/cm^2^), the mask performs an appropriate role as an immobilization tool. Therefore, in this study, we focused on checking whether the mask force is too weak (falls below 100 gf/cm^2^) or falls to ‘0′ to assure the mask is performing an appropriate role as an immobilization tool. However, with existing monitoring equipment, it is not easy to check the compression force inside the mask.

The monitoring system developed in this study can provide information in real time on whether the thermoplastic mask is properly pressing the patient as an immobilization tool. With such information, users can take appropriate actions both in real time and patient-specific level. The GUI of the monitoring system is designed to give a warning signal when there is a problem with the compression force of the mask. For instance, if the yellow light is on, the patient should be observed more carefully, and if the red light is on, a readjustment is recommended.

The ultimate goal of an immobilization tool is complete prevention of patient positioning errors due to mobility. The real-time TMCF monitoring system developed in this study is expected to be of great help in maintaining the clinical utility of the immobilization tool. This study assumed that if it is possible to keep the compression force of the mask consistent throughout the whole treatment course, it would help improve the accuracy of head and neck cancer radiation treatment. In this early study, we verified the feasibility of the system with a cohort of six volunteers only. In further studies, we are planning to investigate the functionality and effectiveness of the system with a cohort of actual patients.

## 5 Conclusion

We developed a real-time TMCF monitoring system. With the developed system, it is possible to monitor the effectiveness of the mask in real time by continuously measuring the compression force between the mask and patient during the treatment. The monitoring system can provide information in real time on whether the thermoplastic mask is properly pressing the patient as an immobilization tool. Although the number of volunteers participating in the study was small, these preliminary results suggest that the system could ostensibly improve the setup accuracy of thermoplastic masks.

## Data Availability

The original contributions presented in the study are included in the article/Supplementary Material; further inquiries can be directed to the corresponding authors.
